# The co-moderating effect of social support and religiosity in the association between psychological distress and coping strategies in a sample of lebanese adults

**DOI:** 10.1186/s40359-023-01102-9

**Published:** 2023-03-06

**Authors:** Daniella Mahfoud, Mirna Fawaz, Sahar Obeid, Souheil Hallit

**Affiliations:** 1 Research Department, Psychiatric Hospital of the Cross, Jal Eddib, Lebanon; 2 Faculty of Health Sciences, Beirut Arab University, Tareek Al Jadida, Afeef Al Tiba, Beirut, 1105 Lebanon; 3 Social and Education Sciences Department, School of Arts and Sciences, Lebanese American University, Jbeil, Lebanon; 4 School of Medicine and Medical Sciences, Holy Spirit University of Kaslik, P.O. Box 446, Jounieh, Lebanon; 5 Applied Science Research Center, Applied Science Private University, Amman, Jordan

**Keywords:** Coping strategies, Psychological distress, Religiosity, Social support, Lebanon

## Abstract

**Background:**

Coping involves attempts to mitigate the negative repercussions of stressful situations including psychological distress. The aim of this study was to assess factors affecting coping and examine the role of social support and religiosity in moderating the association between psychological distress and coping strategies in a sample of Lebanese adults.

**Methods:**

A cross-sectional study was carried out between May and July 2022, enrolling 387 participants. The study participants were asked to complete a self-administered survey containing the Multidimensional Scale of Perceived Social Support Arabic Version, the Mature Religiosity Scale, the Depression Anxiety Stress Scale, and the Coping Strategies Inventory-Short Form.

**Results:**

Higher levels of social support and mature religiosity were significantly associated with higher problem- and emotion-focused engagement scores and lower problem- and emotion-focus disengagement scores. In people experiencing high psychological distress, having low mature religiosity was significantly associated with higher problem-focused disengagement, seen at all levels of social support. In people experiencing high psychological distress, having moderate mature religiosity was significantly associated with higher problem-focused disengagement, seen at both moderate and high levels of social support.

**Conclusion:**

Our findings provide novel insight into the moderating effect of mature religiosity in the association between psychological distress and coping strategies affecting adaptive behavior to stress.

## Background

When confronted with social or physical stressors, the organism’s adaptive capabilities and resources are put to the test [1]. Coping therefore attempts to control, regulate, tolerate, decrease, or limit the consequences of these stressors [2]. It is described as the thoughts and strategies that help overcome both internal and external constraints in stressful situations [3]. These various responses that characterize how people behave under stress are referred to as coping strategies [4]. Understanding these coping strategies is essential for explaining their short-term effects on the resolution of stressors as well as their long-term effects on physical and mental well-being. However, there is limited consensus among individuals on a unique pattern of coping [5]. A significant development in the notion of coping has been made by Lazarus and Folkman [6]. They defined two main strategies: problem-focused coping, intending to manage or modify the problem producing the stress, and emotion-focused coping, aiming to regulate the emotional reactions to the problem [6]. Other theories have suggested dividing coping into active, passive, cognitive, and behavioral mechanisms, although strategies that approach and strategies that avoid a stressor are the most common ways of coping [5, 7]. As a result, engagement strategies, which include approach-related actions, lead to confronting stressors, thereby limiting their long-term psychophysiological effects. Whereas disengagement or avoidance strategies aim to limit exposure to stressful experiences, they frequently have positive short-term effects but have long-term consequences, including depressive symptoms [8]. Therefore, it is quite important to detect factors that affect the general tendency of the person to engage in one coping strategy or another, while the aim is always to facilitate the individual’s adjustment and reduce the negative effects of stressful stimuli.

Coping is quite contextual; it must evolve over time in response to various stressful situations in order to be effective. As a result, a shift in a person’s primary coping mechanisms can be seen over time [9], and it is dependent on many circumstances and factors. Personality qualities or static environmental features may impact the coping mechanisms adopted [9]. However, significant individual variability exists, and numerous sociodemographic and clinical factors appear to alter the pattern of coping mechanisms [10]. In terms of psychological parameters, greater anxiety scores were related to emotion-focused coping strategies, whereas higher levels of depression were associated with more frequent employment of the escape-avoidance strategy [11]. Psychological distress, which entails high levels of stress, anxiety, and depression [12], was also shown to be associated with coping styles. Previous studies have shown that adoption of problem-focused strategies is positively associated with psychological distress [13]. Behavioral escape-avoidance was the most important coping strategy leading to general psychological distress, followed by cognitive escape-avoidance [14]. Besides, another study has shown that among coping strategies, behavioral disengagement, self-blame, and venting were significantly associated with psychological distress, while humor and positive reframing were negatively associated with it [15].

In addition to psychological distress, social support, regarded as measures implemented for a person by their social connections [16], has been shown to play a protective role when facing stressors [17, 18]. It was previously shown that identifying and employing social support and building beneficial interactions with the surrounding network could facilitate the coping process by developing the coping abilities of the person [19]. Besides, psychological distress is increased by a lack of social support from one’s spouse, family, and friends [20], while enhancing resistance to stress is possible through good social support [21]. Increased social support reduces psychological distress [22], because sharing concerns with others makes people feel better and prepares them to deal with any difficult situation or stress [23]. The association of social support with both psychological distress and coping suggests that it may play a role in moderating the association between these two. Religiosity is another potential moderator, as it has been identified as a frequently adopted strategy to cope during stressful situations [24]. In the literature, religion had an impact on mental health; there was a correlation between religiosity and fewer depressive symptoms when faced with stressful situations [25]. Religiosity was suggested to be associated with psychological distress, but it was even more relevant in terms of psychological well-being [26]. Several studies have shown that religion can help people cope with a wide range of personal and social challenges [27–29]. This can be a result of the positive association between religion and cognitive coping processes, which are tangentially linked to increased wellbeing and less psychological suffering [27]. Therefore, reduced stress perception has been linked to effective religious coping mechanisms [30]. Previous findings indicate that coping self-efficacy mediates the protective effect of religious coping against psychological distress [31]. As a result, the evidence reveals that psychological distress, religiosity, and social support all have an impact on the coping mechanisms adopted. Therefore, we suggest in this study that social support and religiosity mutually moderate the relationship between psychological distress and coping strategies.

The Lebanese population have recently experienced a number of very distressing events and disasters. People were prompted to begin a massive revolution as a result of the country’s situation, which sparked public turbulence as well as political and social instability. This disturbance then led the financial system to immediately collapse, bringing the nation even closer to bankruptcy, and the unemployment rate became extremely high [32]. Besides, the COVID-19 pandemic’s emergence has been shown to be detrimental to people’s psychological wellbeing, raising stress, anxiety, and depression levels [33]. The pandemic and the nation’s deteriorating socioeconomic conditions were having a significant cumulative impact on the mental health of its people. Then followed the Beirut explosion, which exacerbated Lebanon’s economic and political conditions. In particular, this had a significant impact on people’s mental health and living situations [34]. The current crisis is the worst in its history, leading to an increase in general psychological distress and suffering. However, Lebanese individuals appear to be able to cope with trauma [35]. During difficult times, people frequently turn to spirituality for support and consolation [36]. And in Lebanon, religion occupies a significant portion of social and political life; the majority of Lebanese view God as omnipotent, the ultimate determiner, and the source of miracles [37]. Furthermore, how individuals deal with adversity is determined by the level of social support they receive [38], and Lebanese typically have a large supportive social network. Therefore, in light of the increasing collapse of the health, political, and economic sectors, it is crucial to shed light on the factors that affect the coping mechanisms that enable individuals to adjust and retain their wellness. Given this, the current study’s objectives were to evaluate the factors that impact the adoption of coping strategies and explore the moderating roles that social support and religion have in the association between psychological distress and coping strategies.

## Methods

### Study design

A cross-sectional study was carried out between May and July 2022 and enrolled 387 participants. Due to the social restrictions imposed by the COVID-19 pandemic, we used an anonymous, self-administered survey created on Google Forms. The link was shared among the participants and sent to all districts/governorates of Lebanon (Beirut, Mount Lebanon, North Lebanon, South Lebanon, and Bekaa) through social networks, using the snowball sampling method technique. All participants above the age of 18 were eligible to participate and were asked to send the link to other subjects. Excluded were those who refused to fill out the survey.

### Minimal sample size calculation

We used G*Power software to determine the sample size. The minimum required sample size was 226 participants, considering an alpha error of 5%, a power of 90%, a minimal model r-square of 10% and allowing 15 predictors to be included in the model.

### Survey

The first part of the survey included an explanation of the study topic and objective and a statement ensuring the anonymity of respondents. The participant had to select the option stating “*I consent to participate in this study”* to be directed to the survey.

The second part of the survey contained sociodemographic information about the participants (age, gender, region of living, marital status, and education level). The Household Crowding Index (HCI) [39], reflecting the socioeconomic status of the family was also included. It is the ratio of the number of people living in the house over the number of rooms (excluding the kitchen and the bathrooms).

The third part included the following scales used in this study:

### Multidimensional scale of perceived social support arabic version (arabic-MDSPSS)

The Multidimensional Scale of Perceived Social Support is a brief research instrument that aims to measure perceived social support. There are 12 items overall, measuring three subscales of four items each, covering the three dimensions: family, friends, and significant others. On a seven-point Likert scale, each item is assessed (1 = very strongly disagree; 7 = very strongly agree). The results for each item are added together to generate the final score. Higher ratings indicate increased social support [40]. We used the Arabic version already translated and validated in Lebanon [41]. In this study, the Cronbach’s alpha value was 0.97.

### Mature religiosity scale (MRS)

This MRS was developed in order to provide criteria for assessing a person’s level of faith both from a theological and a psychological angle. It evaluates how well a person’s religiosity fits into their everyday routine and how it has changed over time. It has 16 items, each of which is graded on a Likert scale from strongly disagree (0) to strongly agree (4). The range of possible scores is 16 to 80, with a higher score indicating a higher level of religiosity [42]. The Arabic version of the scale has been used previously [43]. In this study, the Cronbach’s alpha value was 0.98.

### Depression anxiety stress scale 8-items (DASS-8)

The DASS-8, a shortened version of the DASS-21, consists of eight items divided into three subscales: depression (3 items), anxiety (3 items), and stress (2 items). Responses to the items are scored on a 4-point scale, ranging from 0 (did not apply to me at all) to 3 (applied to me very much or most of the time). The overall score of the DASS-8 ranges from 0 to 24, whereas the subscale scores range from 0 to 9, 0 to 9, and 0 to 6, respectively. Higher scores equate to a higher level of symptom affirmation [44, 45]. In this study, the Cronbach’s alpha value was 0.90.

### Coping strategies inventory - short form (CSI-SF)

The CSI was initially developed as a 78-item survey designed to classify coping responses according to coping intent and response directionality. This approach classifies individuals based on a 2 × 2 matrix that measures how frequently each strategy is adopted. First, coping strategies are divided into two categories: engagement strategies, which include approach-related acts that lead to addressing stressors, and disengagement strategies, which aim to minimize exposure to unpleasant stimuli. Within each of these categories, the coping attempt is either problem-focused or emotion-focused. The responses of the participants are recorded using a four-point Likert scale. Following a validation study, the original CSI was shortened to a 16-item form, validated in Lebanon [46]. The CSI-SF was designed in the same manner as the original scale, with four 4-item subscales: (a) Problem-Focused Engagement, (b) Problem-Focused Disengagement, (c) Emotion-Focused Engagement, and (d) Emotion-Focused Disengagement [47]. In this study, the Cronbach’s alpha values were as follows: problem-focused engagement (0.83), problem-focused disengagement (0.78), emotion-focused engagement (0.73) and emotion-focused disengagement (0.75).

### Statistical analysis

The SPSS software v.25 was used for the statistical analysis. The coping scores were considered normally distributed since the skewness and kurtosis values varied between − 1 and + 1 [48]. For the bivariate analysis, the Student t was used to compare two means and the Pearson test was used to correlate two continuous variables. Four linear regressions were conducted, taking each coping strategy score as a dependent variable. The absence of multicollinearity was confirmed using the Variance Inflator Factor (VIF) values that were all below 2.5 [49]. The moderation analysis was conducted using PROCESS MACRO v3.4, model 1 taking psychological distress as the independent variable, social support and mature religiosity as moderators and each coping score as the dependent variable. Results of the linear regressions and the moderation model were adjusted over variables that showed a p < .25 in the bivariate analysis. P < .05 was deemed statistically significant.

## Results

### Sociodemographic and other characteristics of the sample

Three hundred eighty seven participants participated in this study, with a mean age of 26.17 ± 11.47 years and 58.4% females. Other descriptive statistics of the sample can be found in Table 1.

**Table 1:** Sociodemographic and other characteristics of the sample (N = 387)

Variable	N (%)
Sex	
Male	161 (41.6%)
Female	226 (58.4%)
Marital status	
Single	311 (80.4%)
Married	76 (19.6%)
Education level	
Secondary or less	66 (17.1%)
University	321 (82.9%)
Region of living	
Urban	294 (76.0%)
Rural	93 (24.0%)
	**Mean ± SD**
Age (years)	26.17 ± 11.47
Household crowding index (persons/room)	1.47 ± 1.00

### Bivariate analysis of factors associated with coping strategies

The results of the bivariate analysis of factors associated with coping strategies are summarized in Tables 2 and 3. The results showed that a higher mean emotion-focused disengagement score was found in participants living in urban compared to rural areas. Moreover, higher social support and mature religiosity were significantly associated with higher problem- and emotion-focused engagement scores and lower problem- and emotion-focused disengagement scores.

**Table 2:** Bivariate analysis of factors associated with coping strategies scores

Variable	Problem-focused engagement (mean ± SD)	*p*	Problem-focused disengagement (mean ± SD)	*p*	Emotion-focused engagement (mean ± SD)	*p*	Emotion-focused disengagement (mean ± SD)	*p*
Sex		0.435		0.361		0.385		0.184
Male	12.86 ± 3.53		10.42 ± 3.33		12.64 ± 3.37		11.21 ± 3.32	
Female	12.58 ± 3.42		10.73 ± 3.24		12.35 ± 3.23		11.65 ± 3.19	
Marital status		0.938		0.865		0.495		0.182
Single	12.69 ± 3.63		10.61 ± 3.30		12.52 ± 3.33		11.37 ± 3.37	
Married	12.72 ± 2.70		10.54 ± 3.21		12.24 ± 3.09		11.86 ± 2.68	
Education level		0.888		0.420		0.369		0.060
Secondary or less	12.65 ± 2.97		10.89 ± 3.13		12.14 ± 3.00		12.15 ± 2.71	
University	12.71 ± 3.56		10.54 ± 3.31		12.54 ± 3.34		11.32 ± 3.34	
Region of living		0.539		0.344		0.199		**0.036**
Urban	12.64 ± 3.37		10.69 ± 3.14		12.35 ± 3.17		11.66 ± 3.16	
Rural	12.89 ± 3.74		10.29 ± 3.70		12.85 ± 3.61		10.85 ± 3.48	

Numbers in bold indicate significant *p* values.

**Table 3:** Correlations of continuous variables with coping strategies scores

	1	2	3	4	5	6	7	8	9
1. Problem-focused engagement	1								
2. Problem-focused disengagement	− 0.57***	1							
3. Emotion-focused engagement	0.62***	− 0.49***	1						
4. Emotion-focused disengagement	− 0.36***	0.47***	− 0.59***	1					
5. Psychological distress	0.27***	− 0.14**	0.39***	− 0.36***	1				
6. Social support	0.46***	− 0.50***	0.24***	− 0.22***	− 0.04	1			
7. Mature religiosity	0.38***	− 0.57***	0.30***	− 0.31***	0.05	0.61***	1		
8. Age	0.02	− 0.02	− 0.02	0.04	0.02	− 0.03	− 0.03	1	
9. Household crowding index	0.03	− 0.07	− 0.003	0.04	− 0.02	− 0.04	0.07	0.13*	1

*p < .05; ***p < .001

### Multivariable analysis

Higher social support (Beta = 0.07), mature religiosity (Beta = 0.03) and psychological distress (Beta = 0.16) were significantly associated with more problem-focused engagement (Table 4, Model 1) and with less problem-focused disengagement (Beta = − 0.05, − 0.09 and − 0.07 respectively) (Table 4, Model 2). Higher social support (Beta = 0.02), mature religiosity (Beta = 0.05), psychological distress (Beta = 0.21) and living in a rural region compared to urban (Beta = 0.74) were significantly associated with more emotion-focused engagement (Table 4, Model 3). Finally, higher mature religiosity (Beta = − 0.06), living in a rural area compared to urban (Beta = − 0.93), having a university vs. secondary or less level of education (Beta = − 0.97) and more psychological distress (Beta = − 0.19) were significantly associated with less emotion-focused disengagement (Table 4, Model 4).

**Table 4:** Multivariable analyses (ENTER method)

	Unstandardized Beta	Standardized Beta	*p*	95% CI	VIF
**Model 1: Linear regression taking the problem-focused engagement as the dependent variable (R** ^2^ ** = 0.306)**					
Social support	0.07	0.40	**< 0.001**	0.05; 0.09	1.597
Mature religiosity	0.03	0.12	**0.026**	0.003; 0.05	1.598
Psychological distress	0.16	0.28	**< 0.001**	0.11; 0.21	1.010
**Model 2: Linear regression taking the problem-focused disengagement as the dependent variable (R**^**2**^ **= 0.384)**					
Social support	− 0.05	− 0.27	**< 0.001**	− 0.06; − 0.03	1.615
Mature religiosity	− 0.09	− 0.40	**< 0.001**	− 0.11; − 0.06	1.621
Household crowding index	− 0.16	− 0.05	0.229	− 0.42; 0.10	1.016
Psychological distress	− 0.07	− 0.14	**0.001**	− 0.12; − 0.03	1.011
**Model 3: Linear regression taking the emotion-focused engagement as the dependent variable (R**^**2**^ **= 0.254)**					
Social support	0.02	0.13	**0.024**	0.003; 0.04	1.601
Mature religiosity	0.05	0.22	**< 0.001**	0.02; 0.07	1.608
Region of living (rural vs. urban*)	0.74	0.10	**0.031**	0.07; 1.41	1.008
Psychological distress	0.21	0.39	**< 0.001**	0.16; 0.26	1.011
**Model 4: Linear regression taking the emotion-focused disengagement as the dependent variable (R**^**2**^ **= 0.251)**					
Social support	− 0.01	− 0.07	0.212	− 0.03; 0.01	1.615
Mature religiosity	− 0.06	− 0.26	**< 0.001**	− 0.08; − 0.03	1.647
Region of living (rural vs. urban*)	− 0.93	− 0.12	**0.007**	-1.62; − 0.25	1.054
Sex (females vs. males*)	0.41	0.06	0.18	− 0.18; 1.01	1.076
Marital status (married vs. single*)	− 0.10	− 0.01	0.821	− 0.96; 0.76	1.460
Education (university vs. secondary or less*)	− 0.97	− 0.11	**0.038**	-1.88; − 0.05	1.463
Psychological distress	− 0.19	− 0.36	**< 0.001**	− 0.24; − 0.14	1.023

### Moderation analysis with coping strategies scores taken as dependent variables

The details of the moderation analysis are summarized in Tables 5 and 6. In persons who have high psychological distress, having low mature religiosity was significantly associated with higher problem-focused disengagement. This was seen at low (Beta = − 0.11; t= -3.177; p = .002; 95% CI − 0.18; − 0.04), moderate (Beta = − 0.16; t= -4.298; p < .001; 95% CI − 0.23; − 0.09) and high (Beta = − 0.20; t= -3.781; p < .001; 95% CI − 0.31; − 0.10) levels of social support. Furthermore, in persons who have high psychological distress, having moderate mature religiosity was significantly associated with higher problem-focused disengagement; this was seen at moderate (Beta = − 0.08; t= -3.636; p < .001; 95% CI − 0.12; − 0.04) and high (Beta = − 0.12; t= -3.784; p < .001; 95% CI − 0.19; − 0.06) levels of social support.

**Table 5:** Moderation analysis taking psychological distress as an independent variable, social support/mature religiosity as moderators and each coping score as the dependent variable

Moderator	Beta	t	*p*	95% CI
**Model 1: Problem-focused engagement as the dependent variable.**				
Mature religiosity	− 0.003	-1.527	0.128	− 0.01; 0.001
Social support	0.002	1.461	0.145	− 0.001; 0.01
**Model 2: Problem-focused disengagement as the dependent variable.**				
Mature religiosity	0.005	2.827	0.005	0.002; 0.009*
Social support	− 0.002	-1.679	0.094	− 0.005; 0.001
**Model 3: Emotion-focused engagement as the dependent variable.**				
Mature religiosity	− 0.002	-1.264	0.207	− 0.006; 0.001
Social support	− 0.006	− 0.254	0.799	− 0.05; 0.04
**Model 4: Emotion-focused disengagement as the dependent variable.**				
Mature religiosity	0.001	0.177	0.860	− 0.004; 0.004
Social support	0.001	− 0.217	0.828	− 0.003; 0.003

*indicates significant moderation; results adjusted over age, gender, marital status, education level, region of living and household crowding index.

**Table 6:** Conditional effects of the focal factors at values of the moderators

Mature religiosity	Social support	Beta	T	*p*	95% CI
47.21	33.34	− 0.11	-3.177	**0.002**	− 0.18; − 0.04
47.21	52.40	− 0.16	-4.298	**< 0.001**	− 0.23; − 0.09
47.21	71.47	− 0.20	-3.78	**< 0.001**	− 0.31; − 0.10
62.56	33.34	− 0.04	− 0.963	0.336	− 0.11; 0.04
62.56	52.40	− 0.08	-3.636	**< 0.001**	− 0.12; − 0.04
62.56	71.47	− 0.12	-3.784	**< 0.001**	− 0.19; − 0.06
77.91	33.34	0.04	0.797	0.426	− 0.06; 0.15
77.91	52.40	− 0.002	− 0.067	0.947	− 0.07; 0.06
77.91	71.47	− 0.05	-1.652	0.099	− 0.10; 0.01

Numbers in bold indicate significant *p* values.

## Discussion

The present study examined the relationship between religiosity, social support, psychological distress, and coping strategies in a sample of Lebanese adults. We hypothesized that social support and religiosity would moderate the association between psychological distress and coping strategies. This is crucial for understanding the interaction between all these variables and how they affect the choice of a particular coping mechanism. Our results indicate that higher levels of social support are significantly associated with more problem- and emotion-focused engagement and less problem -focused disengagement coping strategies. Because individuals believe that their social networks contain people who are available and ready to listen, social support may minimize the use of negative disengagement coping mechanisms such as avoidance and increase the use of positive engagement coping mechanisms [50], increasing proactive coping [51]. Previous research has found a positive association between perceived social support and active coping styles but a negative association with avoidant coping styles [52]. However, even though it usually reflects the adoption of an engagement coping strategy, the degree of support and the social setting also have an impact [53]. Furthermore, social support has been shown to protect the well-being of stressed people by increasing their use of problem-focused coping strategies [52]. Regarding religiosity, the results indicate a correlation between higher levels of mature religiosity and higher engagement coping strategies but less disengagement strategies. This association might be explained by the belief that God is in control of the problem [54]; the individual feels more encouraged to face the problem or emotion because they believe that God can alter the situation for the better. It promotes engagement, especially when there are high chances of achieving the established goals, and, to a lesser extent, disengagement, which is more likely to be adaptive, especially under adverse circumstances [55], such as experiencing psychological distress. Psychological distress itself was linked to more problem-focused engagement, more emotion-focused engagement, and less problem- and emotion-focused disengagement. A previous study suggested that adaptive emotion-focused coping and problem-focused coping both had an impact on psychological distress through the mediation of perceived stress [56]. More frequently, psychological distress is associated with disengagement coping strategies [57], and these avoidance-based coping strategies were significantly correlated with depression, anxiety, and stress [58]. However, in our study psychological distress predicted the adoption of engagement or active coping strategies. The participants in our study may have developed psychological resilience, which encouraged them towards positive engagement coping strategies [46].

The moderation analysis revealed that low mature religiosity was substantially linked to more problem-focused disengagement in people who reported high levels of psychological distress. This was observed at all levels of social support. Similarly, moderate mature religiosity was substantially linked to higher levels of problem-focused disengagement in people with high levels of psychological distress, observed at moderate and high levels of social support. In individuals with high psychological distress, in instances of low and moderate mature religiosity, a person appears to avoid the stressor and focus his reactions away from the source of stress by employing a disengagement coping strategy [59]. A lower level of wellbeing and higher distress levels have already been related to a disengaged way of handling stressors [60]. This correlation might have been influenced by levels of religiosity. In a problem-focused coping approach, reduced levels of religious coping have already been linked to avoidant coping strategies such as denying the problem or seeking distraction to avoid directly confronting the root of the problem [61]. Since the least commonly used religious coping strategies are usually passive and negative [28], people with low and moderate mature religiosity tend to adopt a disengagement coping strategy when experiencing significant psychological distress. Because their religious maturity does not give them a greater understanding of the situation, they may just expect God to resolve their problems for them. In our study, the outcome of high psychological distress was problem-focused disengagement, which can be explained by the moderating impact of mature religiosity, which could contribute to being diverted from the stressor rather than confronting it. Despite the fact that the application of particular coping mechanisms is heavily impacted by individual and environmental characteristics, engagement coping mechanisms tend to produce better outcomes and reduce discomfort more than disengagement coping strategies [62]. However, individuals with greater levels of psychological distress were more frequently prone to passive coping [63]. In summary, we assume that the relationship between coping with stressors and increased psychological distress strongly depends on the degree of mature religiosity associated with a particular coping strategy but not much on the social support received. Usually, higher levels of psychological distress are coupled to more disengaged coping mechanisms like passive reaction pattern, palliative reaction, and avoidance [60], and these tend to be affected by how mature a person’s religiosity is.

### Clinical implications

The outcomes of this study emphasize the need for more research into factors that affect the adoption of coping strategies in the general population and for interventions that provide guidance on how to deal with stressful situations that cause psychological distress. Besides, clinicians can try to promote social interaction and support, which can improve psychological well-being and lessen distress [63]. Religious practices may as well lay a solid foundation for individuals to cope positively and actively with various stress factors and circumstances [64], clinicians should also work on encouraging religious reappraisal especially with patients whose religiosity and faith are fundamental to their daily life. This recommends that active coping skills should be taught to the whole public, especially those who are directly impacted by psychological distress, and that people should be encouraged to seek out and maintain religious and social manifestations.

### Limitations

This study carries a number of limitations. Information bias may develop because of the use of self-report measures to evaluate the variables assessed; participants may have overstated or underestimated some questions, introducing subjectivity in responding to questions. The study’s cross-sectional design prohibits us from determining causal relationships between variables and the temporality of occurrences. A selection bias is possible because of the method of recruitment of the participants and the unknown refusal rate. The sample size is small and does not allow generalizability of the results. Finally, because other variables that may influence the adoption of a particular coping strategy were not evaluated in this study, the likelihood of residual confounding bias must be mentioned.

## Conclusion

Our study presents findings that could be of significant clinical relevance in the future, especially with the increase of stressors and factors contributing to severe psychological distress, prompting increased research and interest in coping mechanisms. Overall, our findings highlight the moderating effect of mature religiosity in the association between psychological distress and coping strategies. People experiencing high psychological distress and low mature religiosity were significantly more likely to use problem-focused disengagement, regardless of the level of social support. Additionally, those with high psychological distress and moderate mature religiosity were significantly more likely to use problem-focused disengagement at moderate and high levels of social support. These results provide novel scientific insight into the association between psychological factors affecting adaptive behaviors that impact the coping techniques used to maintain wellbeing and psychological balance, which can be used to guide future research. In light of this study’s findings, future research can focus on checking whether the particular religion followed by the person impacts the coping mechanisms adopted. Besides, examining a different population could help researchers and clinicians better understand the effect of various kinds of stressors on the associations detected since the application of particular coping mechanisms is heavily impacted by individual and contextual variables.

## Data Availability

All data generated or analyzed during this study are not publicly available due the restrictions by the ethics committee (data are owned by the Psychiatric Hospital of the Cross). The dataset supporting the conclusions is available upon request to Ms. Rana Nader (rnader@naderlawoffice.com), a member of the ethics committee at the Psychiatric Hospital of the Cross.
